# Characteristics of pulmonary microvascular structure in postnatal yaks

**DOI:** 10.1038/s41598-021-97760-z

**Published:** 2021-09-14

**Authors:** Ruidong Wan, Ziqi Zhao, Min Zhao, Ke Hu, Jiaxin Zhai, Hongxian Yu, Qing Wei

**Affiliations:** 1 Department of Veterinary Medicine, College of Agriculture and Animal Husbandry, Qinghai University, 251 Ningda Road, Xining, 810016 Qinghai China; 2 College of Eco-Environmental Engineering, Qinghai University, 251 Ningda Road, Xining, 810016 Qinghai China; 3 State Key Laboratory of Plateau Ecology and Agriculture, Qinghai University, 251 Ningda Road, Xining, 810016 Qinghai China

**Keywords:** Developmental biology, Ecology

## Abstract

Yaks are typical plateau-adapted animals, however the microvascular changes and characteristics in their lungs after birth are still unclear. Pulmonary microvasculature characteristics and changes across age groups were analysed using morphological observation and molecular biology detection in yaks aged 1, 30 and 180 days old in addition to adults. Results: Our experiments demonstrated that yaks have fully developed pulmonary alveolar at birth but that interalveolar thickness increased with age. Immunofluorescence observations showed that microvessel density within the interalveolar septum in the yak gradually increased with age. In addition, transmission electron microscopy (TEM) results showed that the blood–air barrier of 1-day old and 30-days old yaks was significantly thicker than that observed at 180-days old and in adults (*P* < 0.05), which was caused by the thinning of the membrane of alveolar epithelial cells. Furthermore, *Vegfa* and *Epas1* expression levels in 30-day old yaks were the highest in comparison to the other age groups (*P* < 0.05), whilst levels in adult yaks were the lowest (*P* < 0.05). The gradual increase in lung microvessel density can effectively satisfy the oxygen requirements of ageing yaks. In addition, these results suggest that the key period of yak lung development is from 30 to 180 days.

## Introduction

The Qinghai-Tibet Plateau has been called the third pole and the roof of the world. The climatic and environmental characteristics of the Qinghai-Tibet Plateau include hypobaric hypoxia, cold, low precipitation and intense ultraviolet radiation conditions. Therefore, many areas are not suitable for human habitation and are even classified as zones unable to sustain life^1^. As a unique and natural geographical region on Earth, the Qinghai-Tibet Plateau has enriched animal resources^2^. Yaks, as a kind of representative plateau animal, are one of the most well-known plateau animals.

Yaks can live in the Qinghai-Tibet Plateau at an altitude of more than 3000 m year-round, this mammal is both very adapted and adaptable to the plateau environment. Some researchers even think that yaks have completely adapted to plateau environments^3^. Many studies have described the biological basis by which yaks have adapted to the high-altitude environment in terms of their anatomy, histology, physiology and molecular biology^4^. Additionally, yaks have many characteristics and attributes that must be regarded as adaptations to higher altitudes which contain low oxygen content in the air. To ensure the faster respiratory frequency of yaks, the trachea in yaks is shorter than that of other cattle, but the diameter is notably larger^5^. Yaks have 14 pairs of ribs, one more than in other cattle^6^, indeed some researchers have reported yaks with 15 pairs of ribs^7^. These additional ribs in the yak make its chest larger than that of other lower altitude dwelling cattle (plain cattle), and yak lungs and hearts are also relatively larger. The increased sizes of these organs in yaks as compared with other cattle help the yak to achieve adequate blood circulation and gas exchange, and therefore enable them to live in high-altitude hypoxic environments^8^. The lungs are the respiratory organs of mammals and the histological structures of yak lungs show many features indicating adaptation to high-altitude hypoxia. The thin-walled pulmonary arteries of yaks may be a feature of adaptation to high-altitude hypoxia^9^. The presence of many elastic fibres in the interalveolar septa and pulmonary vessels in yaks, are beneficial in maintaining lung contraction^10^. The interalveolar septum of yak is also significantly thicker than that of plain cattle. The pneumocyte type I cells, that the blood–air barrier comprises of, have some specialized structures including depressions and vacuoles. The arithmetic and conciliate averages of the blood–air barrier thickness are also notably thinner in yaks than in plain cattle. This thinner blood–air barrier shortens the gas exchange distance and reduces air diffusion resistance, which is beneficial for oxygen acquisition in yak living on the plateau^11,12^.

The biological basis for the yaks’ environmental adaptability is rich and interesting. With the development of modern biotechnology, increasing numbers of researchers are conducting molecular biology research pertaining to the environmental adaptability of this mammal. In the last few years, high-throughput studies on yaks, such as genome and miRNA sequencing and transcriptome analysis, have become popular^13–15^. In fact, there are still many unexplored areas of basic research in this animal. Therefore, in order to obtain additional basic information on yak development, the development of the microvessels within the lungs of postnatal and adult yaks was studied.

## Materials and methods

### Animals

The experimental yaks were divided into four groups: 1-day old, 30-days-old, 180-days-old and adult. Three yaks were selected for each group, regardless of sex, and purchased from a local herdsmen in Haiyan County of Qinghai Province. All of the yaks showed a good nutritional status, and appeared healthy with no apparent diseases or conditions. The yaks were sacrificed by exsanguination in a slaughterhouse. The lungs were obtained immediately after the yak had died, and tissue samples were immediately collected from the diaphragmatic lobe of right lungs (to ensure that obvious blood vessels and the trachea were not gathered). The tissue samples were divided into three parts. One part was cut into 1 cm^3^ sections and fixed with 4% paraformaldehyde (PFA). The other two parts were cut into 1 mm^3^ pieces; one part was fixed with 2.5% glutaraldehyde, and the other was put into a freezing tube and placed into liquid nitrogen.

### Ethics statement

This study was approved by the Institutional Animal Care and Use Committee of Qinghai University (Xining, China). All methods were carried out in accordance with the ARRIVE guidelines and the Animal Ethics Procedures and Guidelines of the People’s Republic of China. No local regulations or laws were overlooked. All yaks used in this study were purchased from local farmers.

### Haematoxylin and eosin staining

Lung tissue samples (1 cm^3^) were fixed in 4% PFA, dehydrated in 30%, 50%, 75%, 95% and 100% ethanol and then treated with xylene before embedding in paraffin. Paraffin-embedded lung tissues were cut into 4 µm sections. The sections were deparaffinized in xylene, and sections were stained either with haematoxylin and eosin (HE) (Y&K Bio, Xi’an, China) or Masson’s trichrome stain, to examine general morphology.

### Immunohistochemistry

The unstained, deparaffinized sections were rinsed with Phosphate Buffered Saline with Twen-20 (PBST) 3 times for 5 min each time. Then, endogenous peroxidase was quenched using 3% peroxide-methanol at room temperature in the dark for 25 min, and then the samples were placed on a decolorizing shaking table 3 times, for 5 min each. The slides were then incubated with 3% foetal bovine serum (Sangon Biotech, Shanghai, China) at room temperature for 25 min. The serum was discarded, and rabbit anti-cattle CD34 and rabbit anti-CD34 polyclonal antibodies (Proteintech group, Wuhan, China) diluted in phosphate buffer saline (PBS) were added. CD34 is a transmembrane glycoprotein known as an angiogenesis marker. The sections were incubated in the primary antibodies overnight at 4 °C. Then, the sections were rinsed in Phosphate Buffered Saline with Twen-20 (PBST) (3 × 5 min), goat anti-rabbit IgG was added, and the sections were incubated for 30 min at 37 °C. 3,3-Diaminobenzidin (DAB) was added to the sections to visualise antibody binding, and the sections were washed 3 times in PBST. Haematoxylin was used to counterstain the nucleus prior to the samples being dehydrated and mounted.

An Olympus BX51 microscope was used to take photomicrographs of the microstructures, images depict 1000× magnification. Transmission electron microscopy.

The TEM lung tissue samples were processed using previously published methods^16^. Fresh lung samples (1 mm^3^) were fixed with glutaraldehyde (2.5%, 24 h) and postfixed with osmium tetroxide (1%, 2 h). The samples were dehydrated in a series of increasing concentrations of ethanol and embedded in Epon812. After preparing semithin sections, ultrathin sections were double stained with uranyl acetate and lead citrate. A 10,000× magnification was used to observe and photograph the sections with a JEM 1230 electron microscope (JEOL, Tokyo, Japan) set at 120 kV.

### Quantitative real-time PCR (qPCR)

The gene expression levels in lung tissues from the yaks in the four age groups were analysed using qPCR. Total RNA was isolated with TRIzol® reagent (Invitrogen, CA, USA). cDNA was obtained by reverse transcription of total RNA using the SYBR PrimeScript RT reagent Kit with gDNA Eraser (Perfect Real Time; Takara, Dalian, China). The forward and reverse primers sequences for the qPCR are shown in Table 1. The genes expression levels were detected using TB Green™ *Premix Ex Taq™* II (TIi RNaseH Plus; Takara, Dalian, China) according to the manufacturer’s instructions. The 2^−ΔΔCT^ method was used to analyse the relative expression of target genes, and the housekeeping gene β-actin was used for normalization.

**Table 1:** Primer sequences.

Primer name	Primer sequence
Vegfa-F	GCCTCCCATTCCCCACTAAATCG
Vegfa-R	CTGCTTGTTTCCAGAATCCGAAG
Epas1-F	TCTTCTATGAGCTGGCGCACGAG
Epas1-R	AGCAAACAGAGGACAGGAGCTTG
β-actin-F	TCACGAAACTACCTTCAATTCCATC
β-actin-R	TTTCTGCATCCTGTTTGCGAT

### Western blot analysis

Equal amounts of proteins of yak lung tissue in different development stages were harvested. These proteins were separated on 10% polyacrylamide gels and transferred onto polyvinylidene difluoride (PVDF) membranes (Sangon Biotech, Shanghai, China). PVDF membranes were blocked in 10% non-fat (skimmed) milk for 3 h and then incubated in rabbit anti-VEGFA polyclonal antibody (OriGene, Maryland, USA) at 4 °C overnight. The membranes were then incubated with a goat anti-rabbit IgG antibody (Abcam, Cambridge, UK) for 2 h being washed 3 times (10 min / time) with Tris-buffered saline with Twen-20 (TBST; containing 0.1% Twen-20). All antibodies were diluted according to the manufacturer’s instructions. Immunoblots were analysed by autograph using a Gel Doc™ XR + Gel documentation system (BIO-RAD, California, USA).

### Statistical analysis

The experimental data are showed as the mean ± standard deviation (SD). The differences between the four groups were compared using one-way ANOVA. *P* values at less than 0.05 were considered significantly different.

## Results

### Microscopic observation of the histological structures in yak lungs

**Figure 1:**
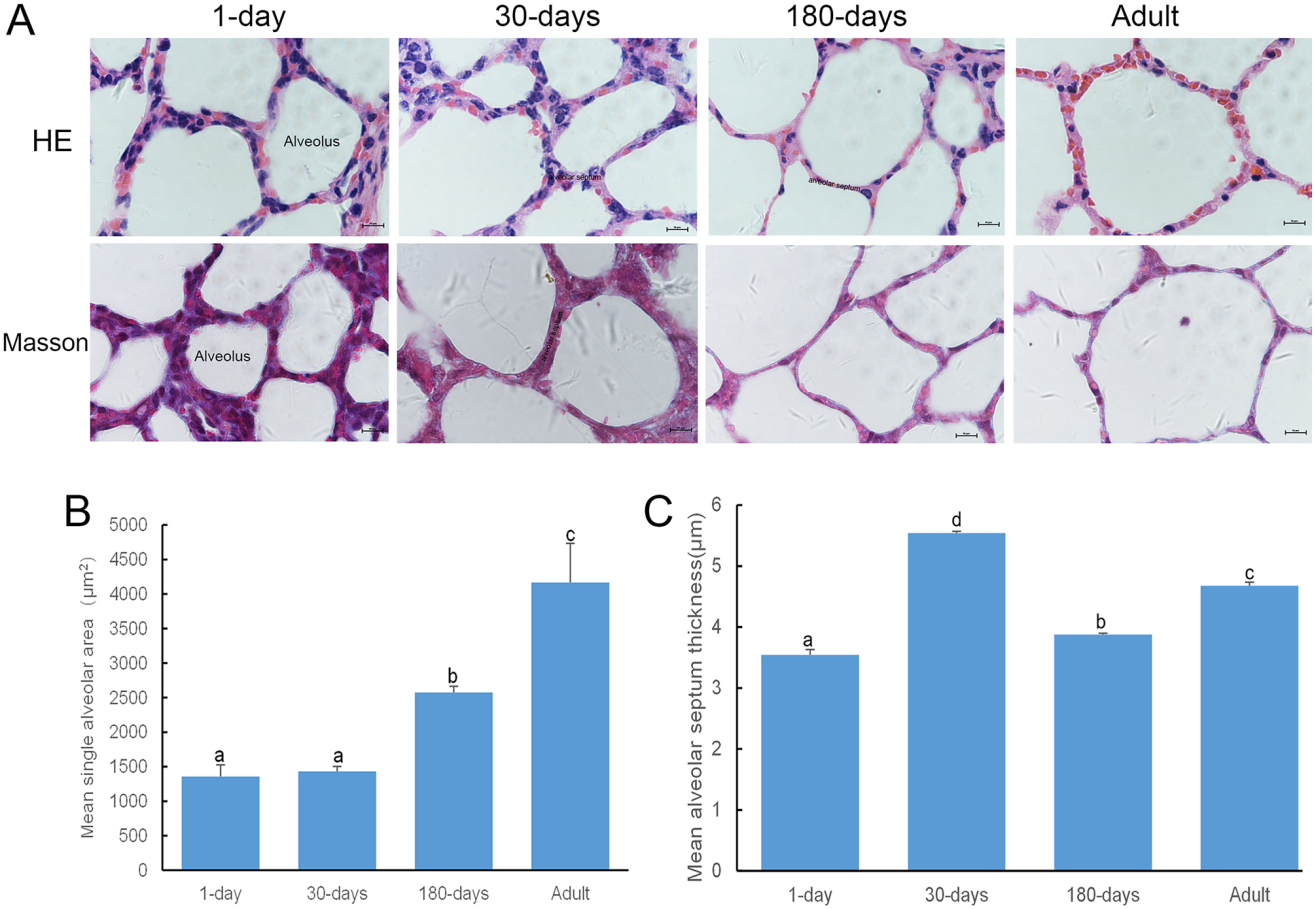
The microstructure of yak alveoli at different developmental stages observed using HE staining and Masson’s staining. (**A**) Representative sections from 1-day old animals to adults (scale bar represents 10 µm, 1000×). (**B**) Graph representing the mean area of individuals pulmonary alveoli (**C**) Graph representing the mean interalveolar septum thickness. Values represent the mean ± SD (n = 3). Different letters (a, b, c, and d) indicate significant differences (*P* < 0.05).

To understand the dynamic development of the yak pulmonary alveolus, pulmonary alveoli were analysed at different developmental stages (1 day, 30 days, 180 days, and adult) using HE and Masson’s staining. As shown in Fig. 1, yaks had fully developed pulmonary alveoli at birth. The areas of each single pulmonary alveolus increased with advancing age, and some translucent membranous structures were observed in the lung tissue in the 180-day and adult yaks. The interalveolar septum was significantly thicker at 30 days than at all other ages, and was filled with cellular components (Fig. 1A). Further measurement analysis found that the area of individual alveoli was significantly larger in the adult group than in the other groups, including those in the 180-day old group. However, the area of the 180-day old group was still higher than yaks aged1 and 30 days (*P* < 0.05; Fig. 1B). The interalveolar septum in adult animals was significantly smaller than observed in 30-day old animals, but it was still significantly larger than measured in the 1-day old and 180-day old animals (*P* < 0.05). Moreover, the interalveolar septum in animals aged 180 days were significantly larger than that of 1-day old animals (Fig. 1C).

### Changes in microvessel density in yak lungs at different developmental stages as evidenced by immunohistochemistry

**Figure 2:**
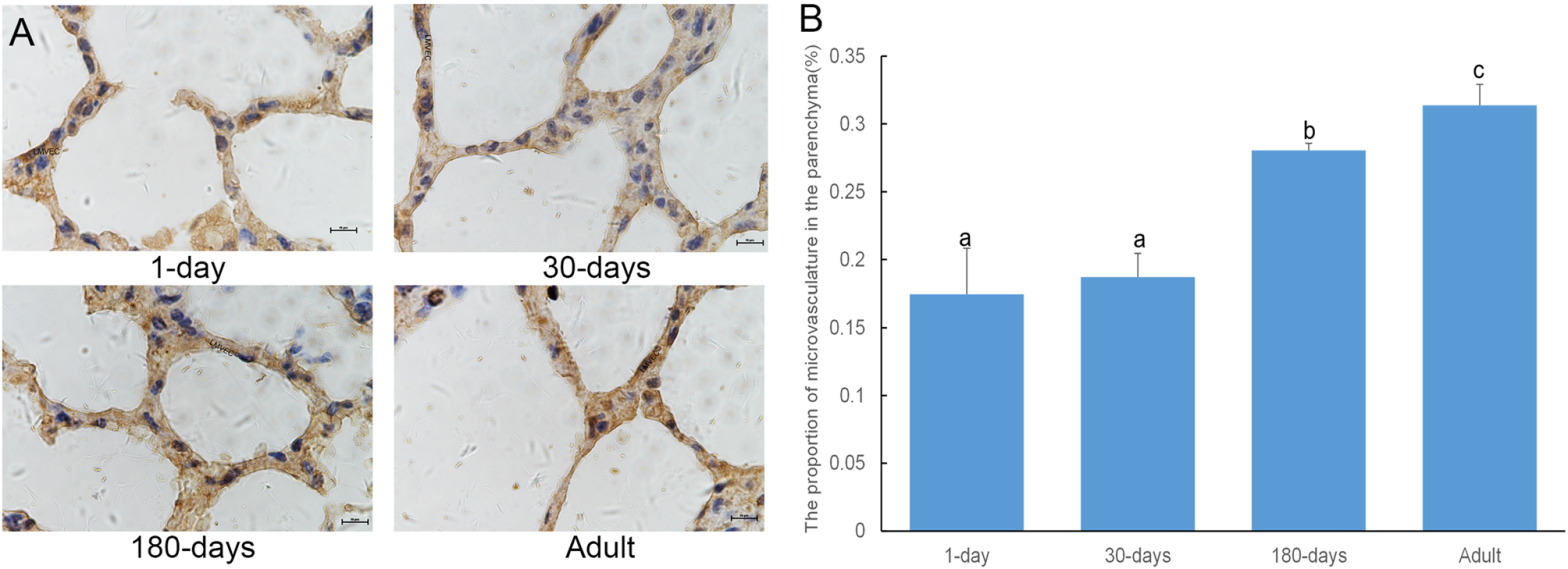
Immunohistochemical staining of yak pulmonary microvascular sections with CD34 at different developmental stages. (**A**) Representative sections from 1-day old animals through to adults (scale bar represents 10 µm, 1000×). Cells stained brown represent positive protein expression results. CD34 was mainly distributed in vascular endothelial cells, showing a high level of expression. (**B**) The graph represents a densitometric analysis of immunohistochemical staining. CD34 content was quantified using Image-Pro Plus software. Values represent the mean ± SD (n = 3). Significant differences are indicated by letters (a, b, c, and d), *P* < 0.05. LMVEC, the lung microvessel endothelial cells.

Immunohistochemical staining with CD34 antibodies enabled observation of microvessel localization in yak lung tissues. Furthermore, the distribution and density of microvessels were assessed (Fig. 2). Immunohistochemical staining showed abundant microvessels in yak lung tissues at all ages (cells stained brown represent positive protein expression results; Fig. 2A). Optical density analysis in the adult group was significantly higher than that measured in the younger age groups (*P* < 0.05). The staining in the 180-day old group was significantly lower than that quantified in the adult group but was still higher than the 1-day old and 30-day old groups (*P* < 0.05). There was no difference between the 1-day old and 30-day old groups (*P* > 0.05; Fig. 2B).

### The ultrastructure of the blood–air barrier of yaks at different developmental stages was observed by TEM

**Figure 3:**
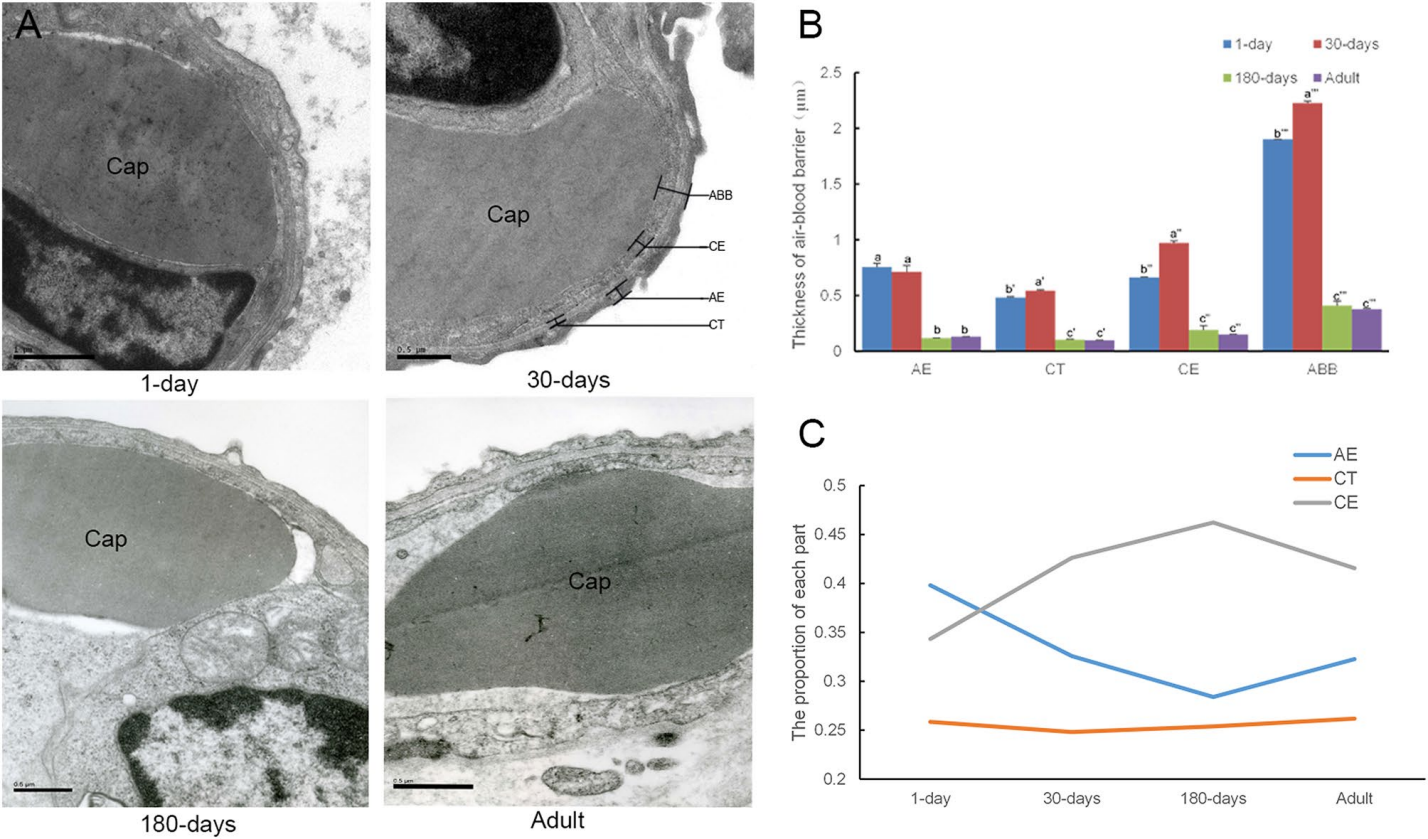
Images of the blood–air barrier at different developmental stages under TEM (scale bar represents 0.5 µm, 10,000×). (**A**) Cross-sectional view of the blood–air barrier in 1-day old, 30-day old, 180-day old, and adult animals under TEM. (**B**) The graph represents measurement analysis of the ABB. (**C**) The graph represents the proportions of AE, CT and CE. As age increased, the CE gradually increased, whilst the AE gradually decreased, and the CT showed almost no change. TEM, transmission electron microscopy; Cap, capillary; AE, alveolar epithelium; CT, connective tissue; CE, capillary endothelium; ABB, blood–air barrier.

The development of the yak air–blood barrier (ABB) and the dynamic changes during the development of the alveolar epithelium (AE), connective tissue (CT) and capillary endothelium (CE) were studied. The pulmonary alveolus was studied at different developmental stages (1 day, 30 days, 180 days, and adult) using TEM (Fig. 3A). The alveolar epithelial cells in the yak mainly comprised of alveolar type I epithelial cells and alveolar type II epithelial cells. The capillary basement membrane in the yak lung was intact, mainly continuous capillaries. The results showed that the ABB was significantly thicker at 30 days than at the other stages (*P* < 0.05). The ABB of the 1-day old group was significantly thinner than that of the 30-days old group (*P* < 0.05) but was thicker than in both the 180-day old and adult groups (*P* < 0.05). However, there were no significant differences between the 180-day old and adult groups (Fig. 3B). At 1 day, the blood–air barrier of the yaks was primarily composed of the AE, followed by the CE and CT. The CE increased and exceeded the AE at 30 days, it reached its maximum proportion at 180 days, and then slightly decreased again in adults. In contrast, the AE decreased at 30 days, reached its minimum portion at 180 days, and then slightly increased again in adults, however it remained lower than the CE. Analysis of the CT showed that from day 1 through to adulthood, this tissue type always had the lowest percentage of the three noted, and showed almost no change over time (Fig. 3C).

### VEGFA and EPAS1 relative expression levels in the lung tissues of plateau yaks at different developmental stages

**Figure 4:**
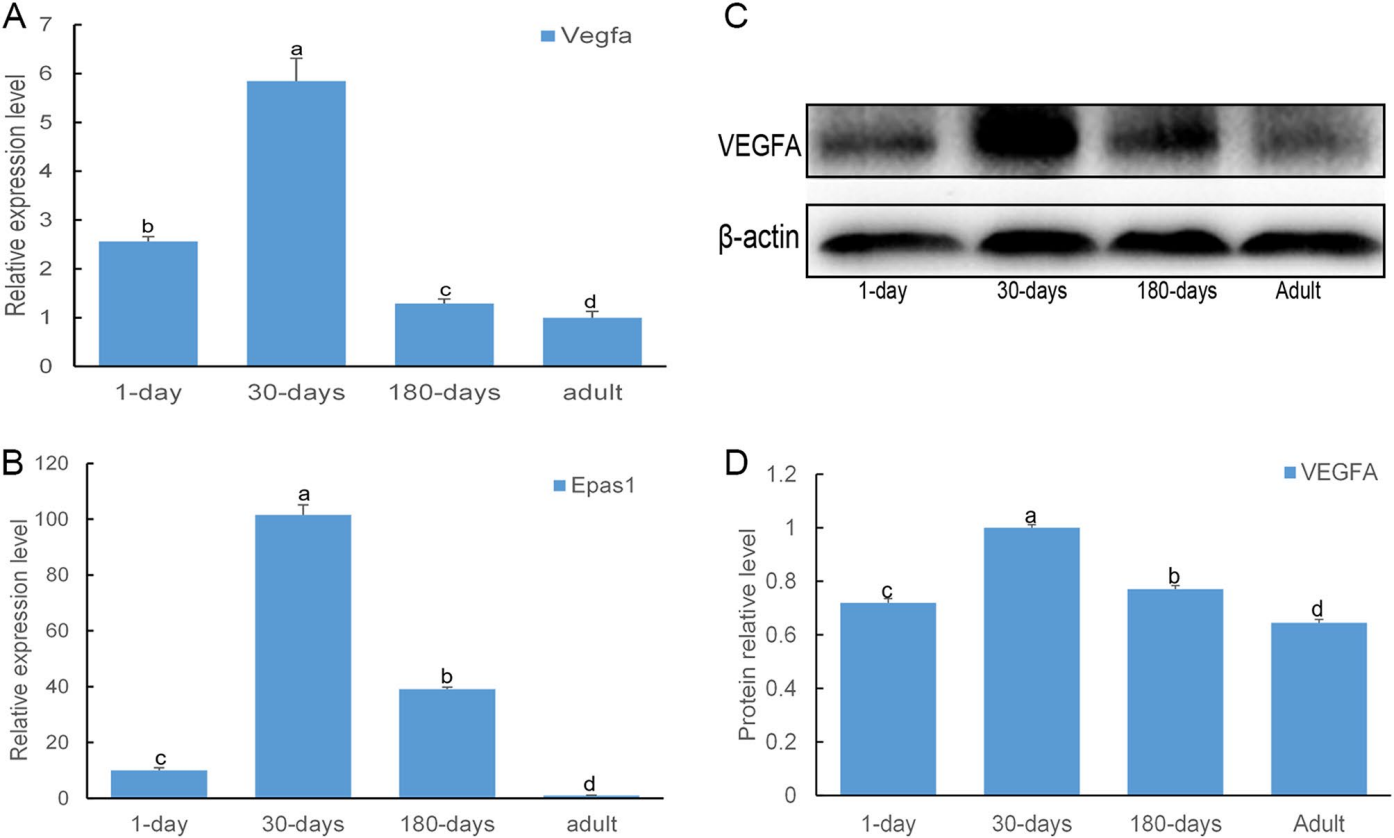
Relative expression levels of VEGFA and EPAS1 in the plateau yak lung tissues at different developmental stages (1 day, 30 days, 180 days, adult). (**A**) Relative VEGFA mRNA levels in the lungs of plateau yaks at different developmental stages. (**B**) Relative EPAS1 mRNA levels in the lungs of plateau yaks at different developmental stages. (**C**) VEGFA protein expression at different developmental stages as determined by western blot analysis. (**D**) The graph represents a densitometric analysis of VEGFA protein expression. (Full-length blots are presented in Supplementary Figures S1 and S2). VEGFA protein content is expressed as relative levels and was quantified using ImagePro Plus software. VEGFA protein expression levels were normalized to β-Actin protein levels. All values above represent the mean ± SD (n = 3). Different letters (a, b, c, and d) indicate significant differences (*P* < 0.05).

To clarify the molecular mechanism of the microvascular changes observed in yak lung tissue after birth, the expression levels of VEGFA and EPAS1, which are related to angiogenesis, were observed using qPCR (mRNA levels) and western blot (protein levels). As shown in Fig. 4, the mRNA and protein expression levels of *Vegfa* and *Epas1* in the yak lung were different at varying developmental stages. The *Vegfa* and *Epas1* mRNA levels were measured by qPCR and Interestingly, the change in the mRNA levels of both *Vegfa* and *Epas1* were similar. The levels of both genes were significantly higher in 30 days old yak lung tissues than at the other stages. The levels in the 1 and 180 days old animals were significantly lower than those in 30-day old animals, yet significantly higher than those expressed in adults. However, the expression levels of *Vegfa* and *Epas1* mRNA were different at 1 day and 180 days. The *Vegfa* and *Epas1* expression levels were significantly higher at 180 days than at 1 day. In contrast, the *Vegfa* mRNA levels at 1 day were significantly higher than those at 180 days (Fig. 4A,B). The expression levels of VEGFA protein in yak lungs at the different developmental stages detected by western blotting were consistent with the qPCR results (Fig. 4C,D).

## Discussion

When studying the postnatal development of Holstein cattle, it was found that the lung tissue entered a rapid tissue development stage after 30 days^17^. In our experiment, it was also observed that the thickness of the interalveolar septum was the largest at the age of 30 days in yaks. Additionally, the number of cells in the alveolar septum increased significantly at this stage and then decreased significantly with advancing age, which suggested that the rapidly increasing cell volume in the interalveolar septum of 30-days old yak may be reserved for rapid lung tissue development in the later stages.

Microvessels are the histological structural basis for blood–air exchange in the lungs^18^. It has been reported that lung tissue microvessel density in plateau animals is significantly higher than that of the same animal species residing in the plains. This increase in microvessel density is conducive in enabling plateau animals to obtain sufficient oxygen and thus ensures better adaptation to the hypoxic plateau environment^19^. There are a large number of microvessels around the pulmonary alveolus to enable gas exchange with the environment. Immunohistochemical staining with CD34, a widely known angiogenesis marker, revealed many CD34-positive cells around the pulmonary alveolus, the number of which increased with age. This finding indicates that pulmonary microvessels gradually increase with age (Fig. 2A), which can allow the yak, which has increased oxygen consumption with age, to better adapt to hypoxic environments. In the 180-day old and adult yak lung tissues, some translucent membrane structures were seen via HE staining, these may be due to the rich expression of some capillaries (Fig. 1A).

Previous publications have shown that the blood–air barrier thickness increased as weight increases, in animals in general^10,20^. However, the present study showed that the thickness of the blood–air barrier in yaks decreased with increasing age and volume, which was mainly caused by thinning of the alveolar epithelium (Fig. 3C). This thinner membrane structure may help to reduce the resistance to air transmission and rapid gas exchange, thereby helping the yak obtain sufficient oxygen during development in the hypoxic environment of a plateau.

*Vegfα* and *Epas1* can promote endothelial cell regeneration and angiogenesis^21,22^. VEGF binds to receptors on endothelial cells and acts as a direct angiogenesis inducer, promoting endothelial cell proliferation and increasing vascular permeability^22^. In vitro experiments have confirmed that VEGF is a direct target of EPAS1 and can regulate NOTCH1, Ang2 and DLL4 to promote the vascular formation of endothelial cells. EPAS1 is preferentially expressed in vascular endothelial cells, and is highly similar to hypoxia inducible factor-1 (HIF-1). In a mouse wound healing model, the adenovirus-mediated transmission of the EPAS1 gene significantly induced the expression of VEGF, FLT-1, FLK-1 and Tie2 mRNA at the wound site, and angiogenesis was promoted. In addition, the proportion of neovascularized wall cells in the EPAS1 treatment group was significantly higher than that in the VEGF treatment group. In summary, the EPAS1 and VEGF genes participate in the development of mature blood vessels and play important roles in angiogenesis. In the present study, expression of *Vegfα* and *Epas1* in yaks was relatively high at birth, reached their maximum levels at 30 days, then gradually decreased with their lowest expression observed in in adults (Fig. 4A,B). There were few pulmonary microvessels in 1-day old and 30-day old yaks. With the increased body size and activity in older yaks, additional microvessels are needed in the alveoli. *Vegfα* and *Epas1* are expressed in the early postnatal period, allowing yaks to generate additional blood vessels to adapt to hypoxia. These results were consistent with our histological results. We speculated that 30 days of age is a critical period of yak lung tissue development. At this time point, the number of cells in yak lung tissue was extremely high. The high expression levels of *Vegfα* and *Epas1* further support this conclusion. In addition, some studies have shown that *Epas1* can regulate the expression of *Vegfα* under hypoxia^23^. As evidenced by the expression trends of these two genes in yak lung tissue at different developmental stages, the expression trends of the two genes were also consistent.

## Supplementary Information

Supplementary Figures.

## Data Availability

All data generated or analysed during this study are included in this published article.

## References

[1] Yu C (2012). Ecological and environmental issues faced by a developing Tibet. Environ. Sci. Technol..

[2] Wang X (2015). Cenozoic vertebrate evolution and paleoenvironment in Tibetan Plateau: Progress and prospects. Gondwana Res..

[3] Sakai A (2003). Cardiopulmonary hemodynamics of blue-sheep, Pseudois nayaur, as high-altitude adapted mammals. Jpn. J. Physiol..

[4] Leslie JDM, Schaller GB (2009). Bos grunniens and Bos mutus (Artiodactyla: Bovidae). Mamm. Species.

[5] Xi O (1991). The adaptation of yak to special ecological environment. China J. Southwest Natl. Coll. (Nat. Sci.).

[6] Zhang H (1987). Yak has 14 pairs of ribs. Chin. J. Vet..

[7] Luo G (1987). Found yak with 15 pairs of ribs. Prataculture&Animalhusbandry (China).

[8] Cui G, Tiancai Lu, Li H, Lai M, Cheng Z, Xia L (1991). Anatomical characteristics of yak heart and lungs. Chin. yak.

[9] Heath D, Williams D, Dickinson J (1984). The pulmonary arteries of the yak. Cardiovasc. Res..

[10] Chen Q, Feng X, Jiang S (2006). Structural study on Plateau adaptability of yak lung. Sci. Agric. Sin. (China).

[11] Wei Q (2006). Observation and comparison of alveolar tissue structure of highland yak. Heilongjiang Anim. Husb. Vet. Med. (China).

[12] Meban C (1980). Thickness of the air–blood barriers in vertebrate lungs. J. Anat..

[13] Xie X (2018). Accumulation of deleterious mutations in the domestic yak genome. Anim. Genet..

[14] Lan D (2018). Transcriptome profile and unique genetic evolution of positively selected genes in yak lungs. Genetica.

[15] Zhang Q (2018). Comprehensive analysis of microRNA(−)messenger RNA from white yak testis reveals the differentially expressed molecules involved in development and reproduction. Int. J. Mol. Sci..

[16] Yang B (2010). Morphological analysis of the lung of neonatal yak. Anat. Histol. Embryol..

[17] Castleman WL, Lay JC (1990). Morphometric and ultrastructural study of postnatal lung growth and development in calves. Am. J. Vet. Res..

[18] Davies P, Maddalo F, Reid L (1985). Effects of chronic hypoxia on structure and reactivity of rat lung microvessels. J. Appl. Physiol..

[19] Wang X (2008). Characteristics of pulmonary acinus structure in the plateau zokor Myospalax baileyi and plateau pika Ochotona curzniae. Acta Zool. Sin..

[20] Weibel ER (1970). Morphometric estimation of pulmonary diffusion capacity. I. Model and method. Respir. Physiol..

[21] Tian H, Hammer RE, Matsumoto AM, Russell DW, McKnight SL (1998). The hypoxia-responsive transcription factor EPAS1 is essential for catecholamine homeostasis and protection against heart failure during embryonic development. Genes Dev..

[22] Clauss M (2000). Molecular biology of the VEGF and the VEGF receptor family. Semin. Thromb. Hemost..

[23] Pan H (2018). Biosci. Rep..

