# Supplementary figures and images for: Characteristics of pulmonary microvascular structure in postnatal yaks
Source: Sci Rep. 2021 Sep 14;11:18265. doi: 10.1038/s41598-021-97760-z (PMC8440534; doi:10.1038/s41598-021-97760-z)

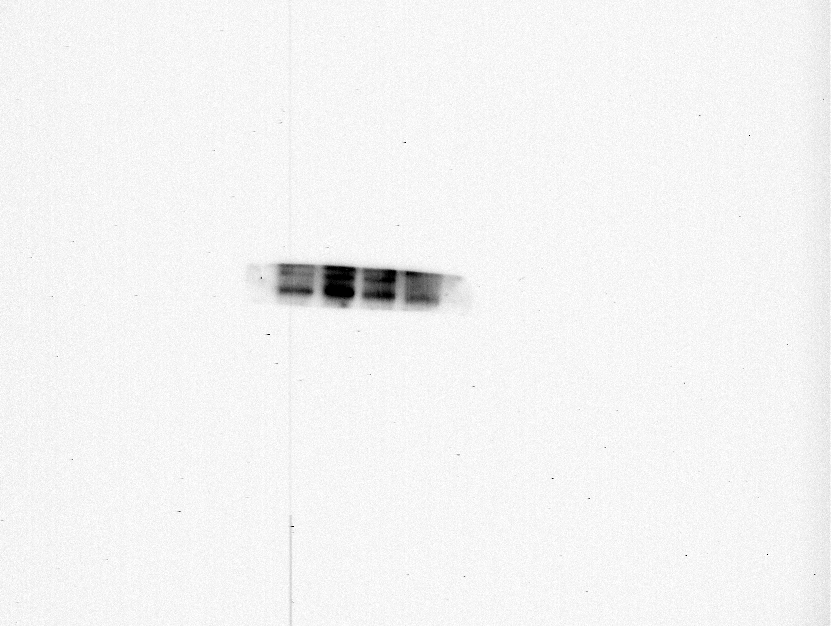


Supplementary Figure S1


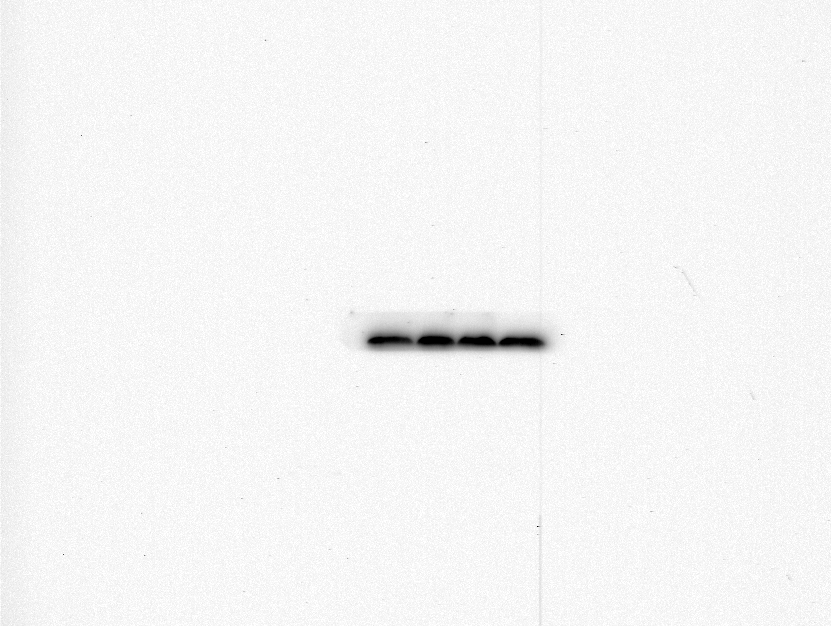


Supplementary Figure S2


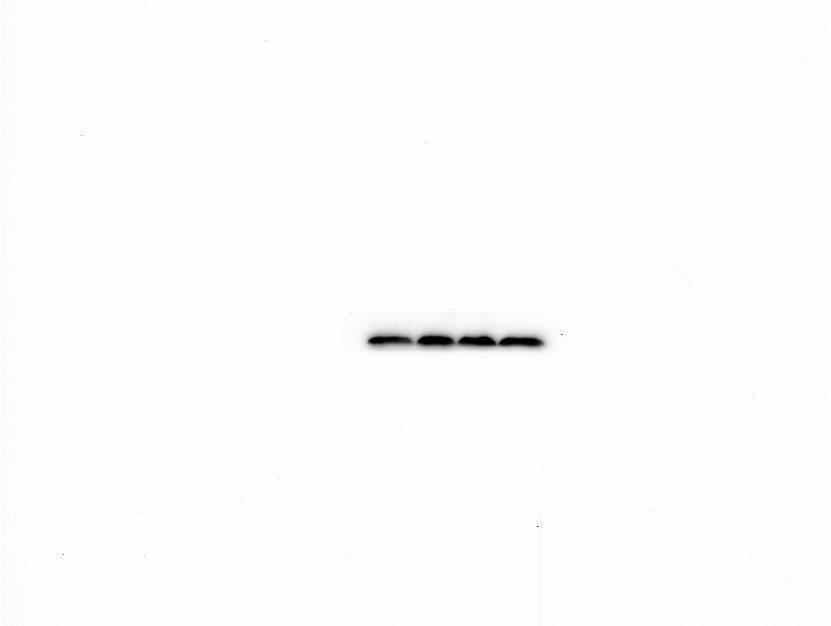

Supplement: Supplementary file 1 — Supplementary Figures. [file 41598_2021_97760_MOESM1_ESM.docx]
